# Supplementary material for: Biochemical properties of chromatin domains define genome compartmentalization
Source: Nucleic Acids Res. 2024 May 29;52(12):e54. doi: 10.1093/nar/gkae454 (PMC11229364; doi:10.1093/nar/gkae454)
Supplement: gkae454_Supplemental_Files [file gkae454_supplemental_files.zip › LuciniEtAl_SupplementaryMaterial_2024-04-21_FF.pdf]

# Biochemical properties of chromatin domains define genome compartmentalization

Federica Lucini[1,8,†], Cristiano Petrini[2,†], Elisa Salviato[2,†], Koustav Pal[2], Valentina Rosti[1,3], Francesca Gorini[1], Philina Santarelli[1], Roberto Quadri[1], Giovanni Lembo[2], Giulia Graziano[2], Emanuele Di Patrizio Soldateschi[1,3], Ilario Tagliaferri[2], Eva Pinatel[3], Endre Sebestyén[2], Luca Rotta[4], Francesco Gentile[5], Valentina Vaira[6], Chiara Lanzuolo[1,3,§,*], Francesco Ferrari[2,7,§,*]

[1] INGM, Istituto Nazionale di Genetica Molecolare "Romeo ed Enrica Invernizzi", Milan, 20122, Italy
[2] IFOM-ETS, The AIRC Institute of Molecular Oncology, Milan, 20139, Italy
[3] ITB-CNR, Institute of Biomedical Technologies, National Research Council, Segrate, 20054, Italy
[4] IEO, European Institute of Oncology IRCCS, Milan, 20141, Italy.
[5] Fondazione IRCCS Ca' Granda-Ospedale Maggiore Policlinico, Milan, 20122, Italy
[6] Division of Pathology, Fondazione IRCCS Ca' Granda Ospedale Maggiore Policlinico, Milan, Italy
[7] IGM-CNR, Institute of Molecular Genetics "Luigi Luca Cavalli-Sforza", National Research Council, Pavia, 27100, Italy
[8] Present Address: Federica Lucini, IFOM-ETS, The AIRC Institute of Molecular Oncology, Milan, 20139, Italy

† Joint first authors
§ These authors jointly supervised this work
* To whom correspondence should be addressed. Email: chiara.lanzuolo@cnr.it; francesco.ferrari@cnr.it

## Supplementary Material

| | |
|---|---|
| **Supplementary Figure 1** | Quality controls on SAMMY-seq experimental procedure |
| **Supplementary Figure 2** | 4f-SAMMY-seq and 10kh-SAMMY-seq recapitulate open and closed chromatin domains |
| **Supplementary Figure 3** | Chromatin fractions read profiles recapitulate epigenetic marks |
| **Supplementary Figure 4** | Relative comparison of chromatin fractions recapitulate heterochromatin regions |
| **Supplementary Figure 5** | 4f-SAMMY-seq based compartments and sub-compartments in mouse C2C12 cells |
| **Supplementary Figure 6** | 4f-SAMMY-seq chromatin fractions and chromatin marks association to chromatin states |
| **Supplementary Figure 7** | Compartments and sub-compartments analysis on low input 4f-SAMMY-seq |
| **Supplementary Table 2** | Comparison between Hi-C, 3f and 4f SAMMY-seq |

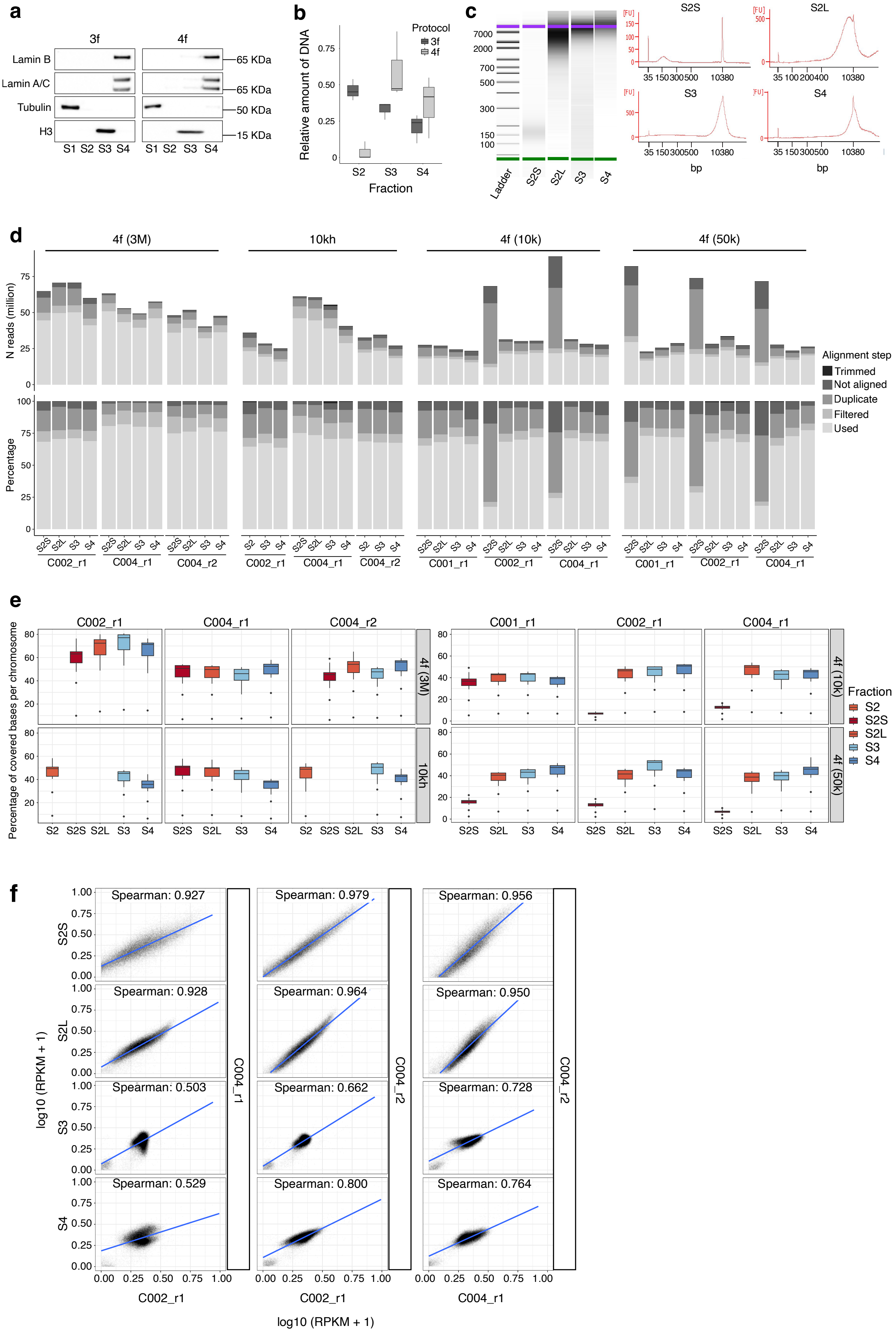

**Supplementary Figure 1 - Quality controls on SAMMY-seq experimental procedure.**
**a)** Representative western blots of chromatin fractions obtained with "3f" and "4f" SAMMY-seq protocols. In both

cases, we find soluble tubulin in S1 fraction, histone H3 in S3 fraction and lamins in S4 fraction.

**b)** Relative abundance (y-axis) of DNA extracted from S2, S3 and S4 fractions, computed as ratio over their sum, in "3f" and "4f" SAMMY-seq protocols (dark and light grey, respectively). In the boxplots, the horizontal lines mark the median, the boxes mark the interquartile range (IQR) and whiskers extend up to 1.5 times the IQR. Relative abundance of DNA was evaluated over 3 independent biological replicates for each protocol.

**c)** Representative bioanalyzer electropherograms of individual 4f-SAMMY-seq fractions, showing the size distribution of DNA fragments before sonication.

**d)** Stacked barplots for the number of sequencing reads obtained for each sample and chromatin fraction, divided in trimmed, not aligned, duplicates, filtered (discarded) and used reads retained for downstream analyses. See also the associated Supplementary Table 1. Individual replicates are reported for 4f-SAMMY-seq experiments on human fibroblasts (3M, 3 million, indicating the starting number of cells), as well as for the 10kh-SAMMY-seq protocol variant, and the 4f-SAMMY-seq scale-down experiments with 10k or 50k starting number of cells.

**e)** Boxplots summarizing coverage across chromosomes for each sample and fraction. The percentage of each chromosome covered by at least one read is reported on the y-axis. In the boxplots, the horizontal lines mark the median, the boxes mark the interquartile range (IQR) and whiskers extend up to 1.5 times the IQR. Individual replicates are reported for 4f-SAMMY-seq experiments, as well as for the 10kh-SAMMY-seq protocol variant, and the 4f-SAMMY-seq scale-down experiments with 10k or 50k starting number of cells.

**f)** Scatter plots of reads distribution profiles (RPKM normalized coverage over 50kb bins) for individual fractions of 4f-SAMMY-seq replicates. Fractions labels are indicated on individual rows (labels on the left) and the replicates compared in each plot are indicated on bottom and right-side axes.

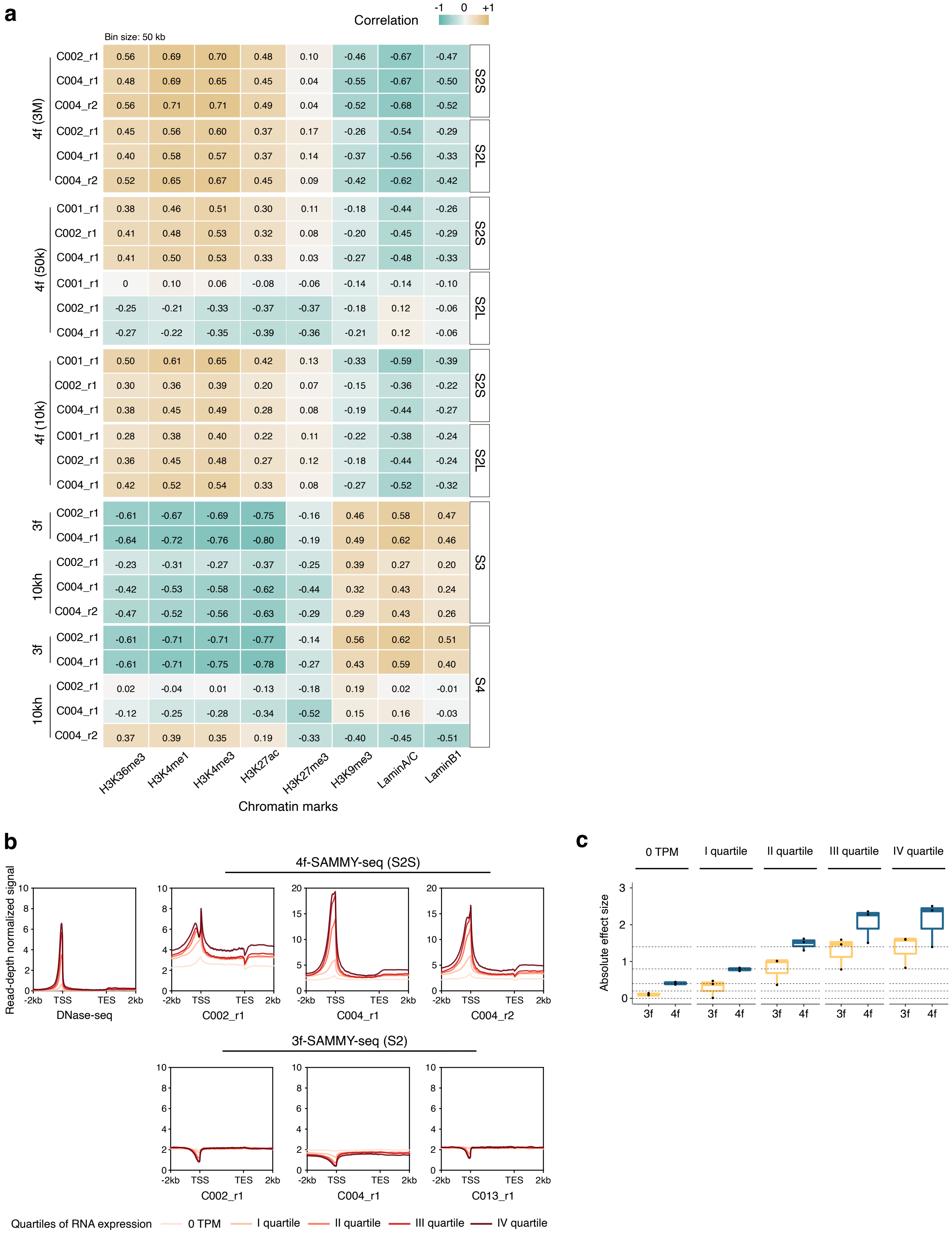


**Supplementary Figure 2 - 4f-SAMMY-seq and 10kh-SAMMY-seq recapitulate open and closed chromatin domains.**

**a)** Genome-wide Spearman correlation between reads distribution profiles for individual selected SAMMY-seq chromatin fractions and ChIP-seq enrichment profiles for histone marks and lamin proteins (x-axis labels) from human fibroblasts. The labels on the left side indicate the protocol versions for 3f-SAMMY-seq (3f), 4f-SAMMY-seq (4f) starting from 3million (3M), 50,000 (50k) or 10,000 cells (10k), and 10kh-SAMMY-seq (10kh). The labels for individual

chromatin fractions (S2S, S2L, S3 and S4) are reported on the right. A row for each replicate is shown and correlation values are reported as numbers and as colour gradient.

**b)** Gene centred meta-profiles for reads distribution profiles of a reference DNase-seq sample (foreskin human fibroblasts from ENCODE, aliases: roadmap-epigenomics:E055) and individual replicates of 4f-SAMMY-seq S2S fraction and 3f-SAMMY-seq S2 fraction. Genes are divided by quartiles of expression level (RPKM) and a meta-profile is drawn for each quartile, as well as for genes with zero RPKM (no reads), as measured by RNA-seq. On the x-axis, the coordinates for the relative genomic position around the rescaled gene body are reported.

**c)** Paired effect size was computed with Cohen's *d* method for the difference in reads coverage at TSS and at an upstream region (-2kb) used as reference background. The absolute effect size value is reported in the boxplot for each replicate (individual dots reported in the plot) and for each expression quartile (labels on top). Data are shown separately for the 3f-SAMMY-seq and 4f-SAMMY-seq experiments (yellow and blue boxes, respectively) from the corresponding panel b. Dashed lines demarcate standard Cohen's *d* cut-offs indicating none, small (>0.2), medium (>0.4), large (>0.8) and very large (>1.4) effect size.

a

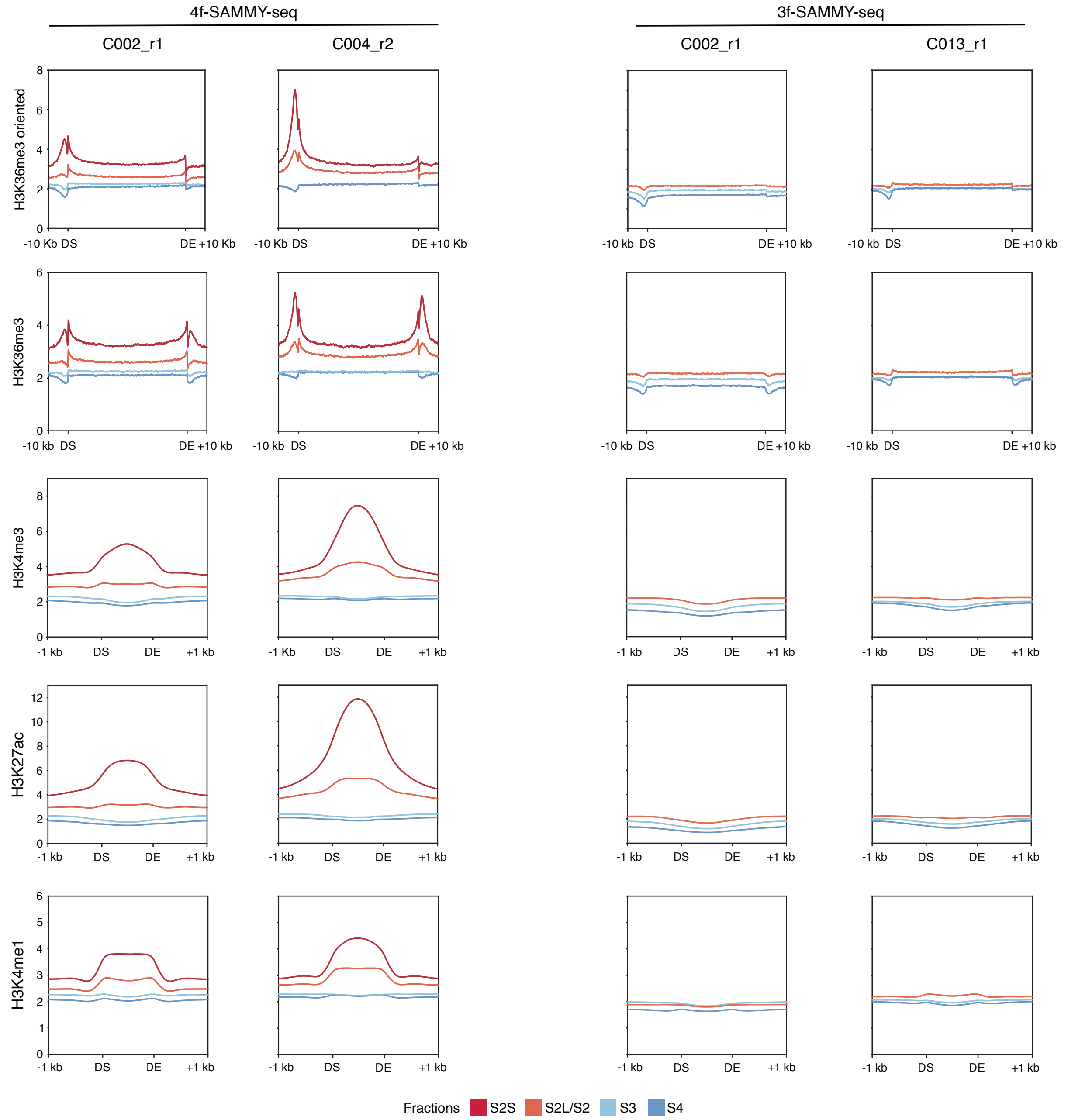


b

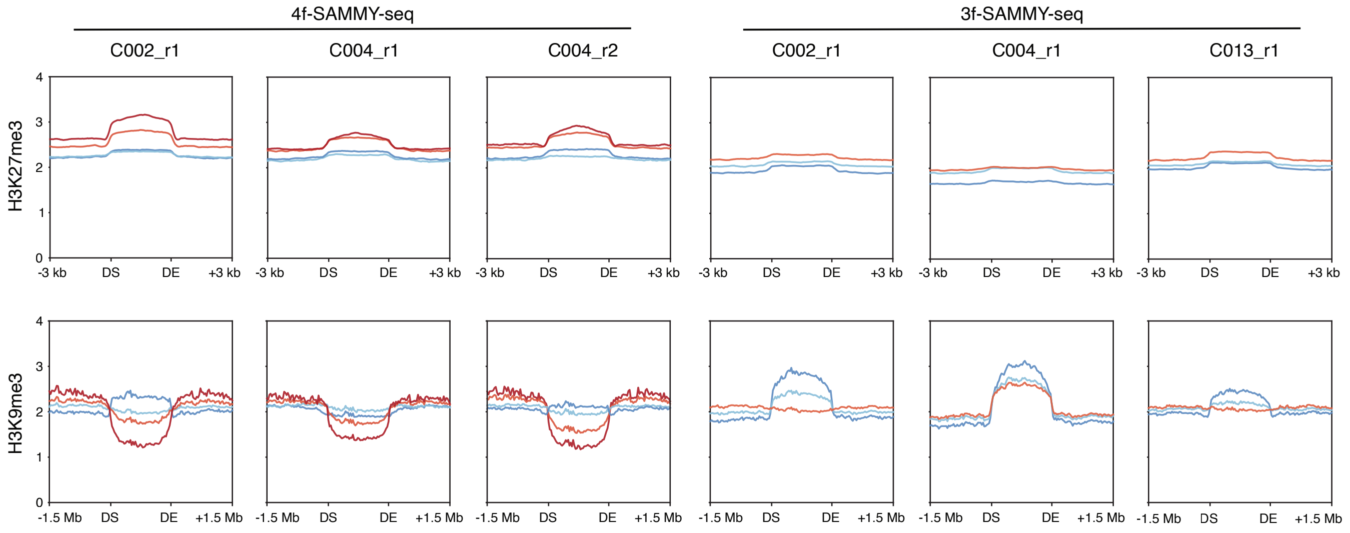


**Supplementary Figure 3 - Chromatin fractions read profiles recapitulate epigenetic marks.**
**a)** Reads distribution meta-profiles for individual "4f" or "3f" SAMMY-seq fractions (labels on the top border). The average reads distribution profiles are computed over chromatin domains marked by enrichment peaks of specific

histone marks (indicated on the left side of each row). The domain start (DS) and domain end (DE) are indicated on the x-axis along with flanking regions coordinates. For H3K36me3 mark, we also reported the meta-profile obtained by orienting the domains according to the corresponding gene's transcribed strand.

**b)** Reads distribution meta-profiles following the same colouring and labelling conventions adopted in panel a, but referring to domains enriched for histone marks associated to inactive chromatin: H3K27me3 and H3K9me3.

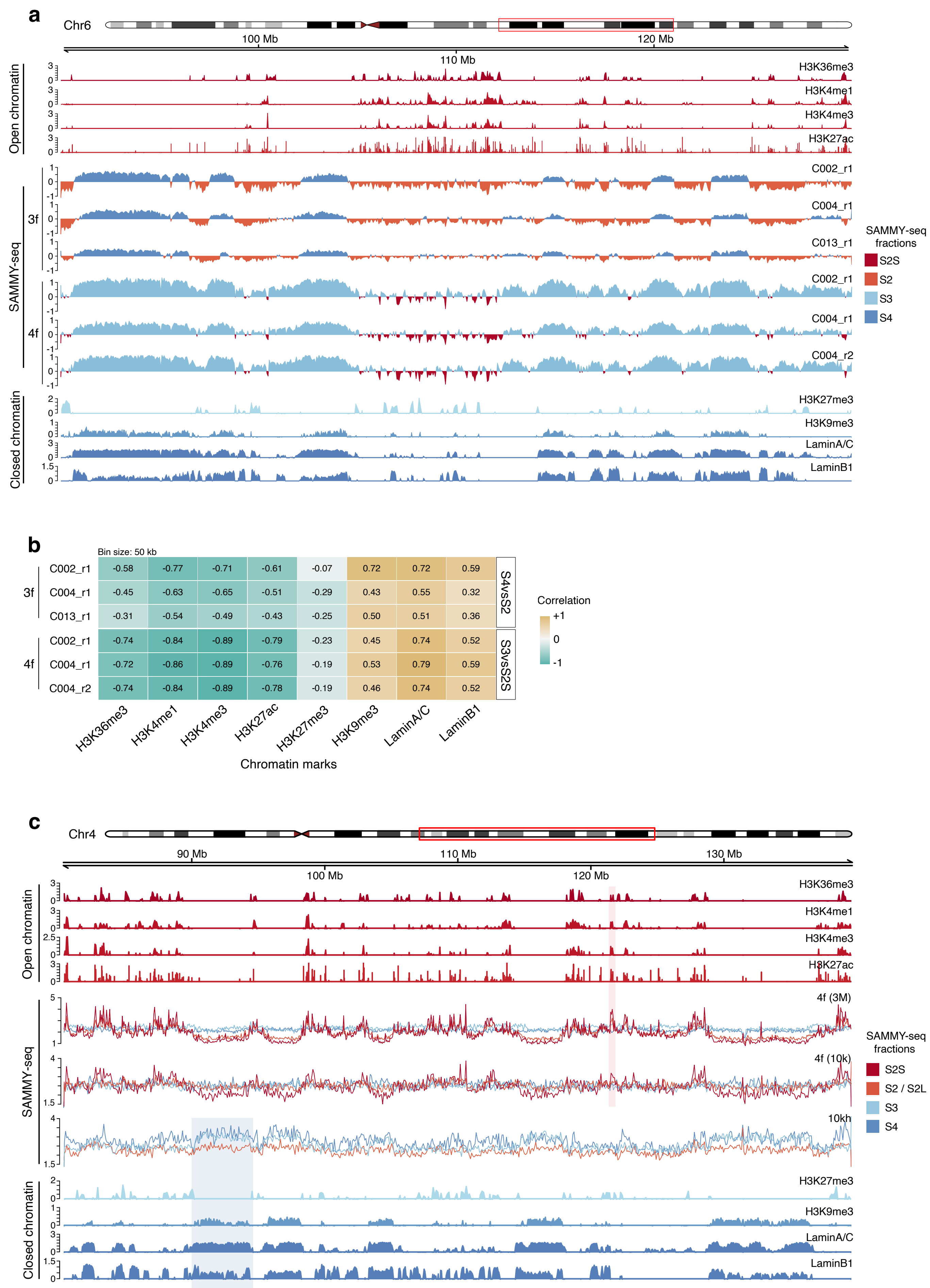


**Supplementary Figure 4 - Relative comparison of chromatin fractions recapitulate heterochromatin regions.** **a)** Representative genomic region (chr6:90,000,000-130,000,000) showing tracks for SAMMY-seq and chromatin marks in human foreskin fibroblasts. From top to bottom: open chromatin marks ChIP-seq enrichment profiles for

H3K36me3, H3K4me1, H3K4me3, H3K27ac; relative enrichment of closed (S3 or S4) over accessible (S2 or S2S) (see color legend) chromatin fractions from "3f" and "4f" SAMMY-seq in all replicates; closed chromatin marks ChIP-seq enrichment profiles for H3K27me3, H3K9me3, Lamin A/C, Lamin B1.

**b)** Genome-wide Spearman correlation between relative enrichment profiles of closed (S3 or S4) over accessible (S2 or S2S) SAMMY-seq chromatin fractions (labels on the right) and ChIP-seq enrichment profiles for histone marks and lamin proteins (x-axis labels) from human fibroblasts. The labels on the left side indicate the protocol versions for 3f-SAMMY-seq (3f) and 4f-SAMMY-seq (4f). A row for each replicate is shown, and correlation values are reported as numbers and as colour gradient.

**c)** Representative genomic region (chr4:80,000,000-140,000,000) showing tracks for SAMMY-seq and chromatin marks in human foreskin fibroblasts. From top to bottom: open chromatin marks ChIP-seq enrichment profiles for H3K36me3, H3K4me1, H3K4me3, H3K27ac; reads distribution profiles for individual fractions of a representative replicate of 4f-SAMMY-seq on 3M (C004_r2) and 10k cells (C001_r1), as well as 10kh-SAMMY-seq (C002_r1); closed chromatin marks ChIP-seq enrichment profiles for H3K27me3, H3K9me3, Lamin A/C, Lamin B1. The shaded areas mark two examples of regions showing enrichment for closed (blue) or open (red) chromatin marks, with corresponding enrichment patterns visible in ChIP-seq as well as in SAMMY-seq fractions.

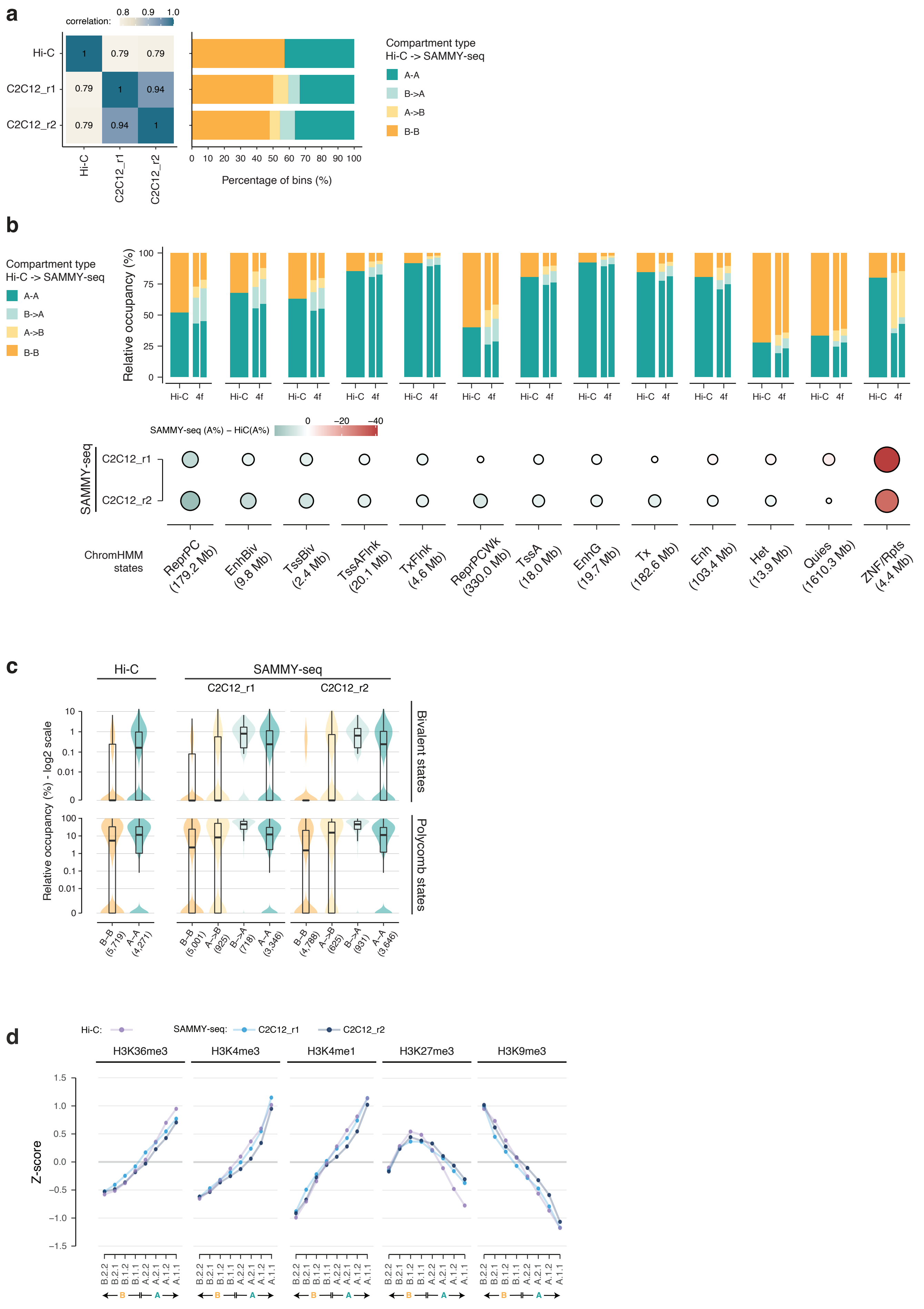


**Supplementary Figure 5 - 4f-SAMMY-seq based compartments and sub-compartments in mouse C2C12 cells.** **a)** Genome-wide pairwise Pearson correlation of chromatin compartments eigenvectors (250kb size bins) defined by Hi-C and 4f-SAMMY-seq in mouse C2C12 cells with individual replicates is reported on the left. For each sample,

the stacked barplot on the right shows the relative distribution (percentage) of genomic bins associated with concordant ("A-A" or "B-B") and discordant ("A->B" or "B->A") compartment classification with respect to Hi-C. The classification is reported using the same colouring and naming convention adopted in Figure 2b.

**b)** Distribution of "A" and "B" compartments (from Hi-C and 4f-SAMMY-seq) across multiple chromatin states from mouse C2C12 myoblasts. The stacked barplots show the percentage of regions associated to concordant ("A-A" or "B-B") and discordant ("A->B" or "B->A") compartments classification for each chromatin state (labels on the bottom margin indicating chromatin states and their total size). The barplot for 4f-SAMMY-seq compartments distribution is divided in two parts to report the specific results for the two replicates (C2C12_r1 and C2C12_r2, respectively). Below the barplot, the dot plot reports for each chromatin state the relative occupancy of 4f-SAMMY-seq vs Hi-C based compartments, computed as the difference in "A" compartment percentage. For each chromatin state (columns, labels and total size for each state at the bottom) positive values (green gradient) indicate a higher percentage of "A" compartment in 4f-SAMMY-seq based classification, whereas negative values (red gradient) indicate a higher percentage of "A" compartment based on Hi-C classification (i.e. higher "B" percentage based on 4f-SAMMY-seq). The size of each dot is proportional to the absolute value in the percentage difference. Chromatin states are ordered from left to right based on the average percentage difference between "A" compartment in 4f-SAMMY-seq replicates (average between the two replicates, represented on the rows) and Hi-C, in a decreasing order.

**c)** Violin and box plots showing for the mouse C2C12 Hi-C dataset and individual 4f-SAMMY-seq replicates (labels on the upper margin) the distribution of Polycomb regulated chromHMM chromatin states. The upper violin plot is for bivalent Polycomb states (TssBiv or EnhBiv) the bottom one for monovalent repressive Polycomb states (ReprPC or ReprPCWk). Each set of violin plots shows the relative occupancy (percentage, y-axis values in $\log_2$ scale) over 250kb genomic bins covering the entire genome and grouped by compartments classification ("A-A", "B-B", "A->B" or "B->A" defined as above) based on individual Hi-C and 4f-SAMMY-seq samples. The number of genomic bins in each group is indicated at the bottom in parentheses (x-axis). In the overlaying boxplots, the horizontal lines mark the median, the boxes margins mark the interquartile range (IQR), and whiskers extend up to 1.5 times the IQR.

**d)** Genome-wide mean ChIP-seq IP over INPUT enrichment in C2C12 cells (centred and scaled by chromosome, see Methods) in the eight sub-compartments classification defined by CALDER. Enrichment for ChIP-seq datasets is shown for sub-compartments obtained using Hi-C (purple points and lines) and 4f-SAMMY-seq data (blue and light blue points and lines for the two replicates). Sub-compartments are sorted from the most compacted (left, B.2.2) to the most accessible one (right, A.1.1).

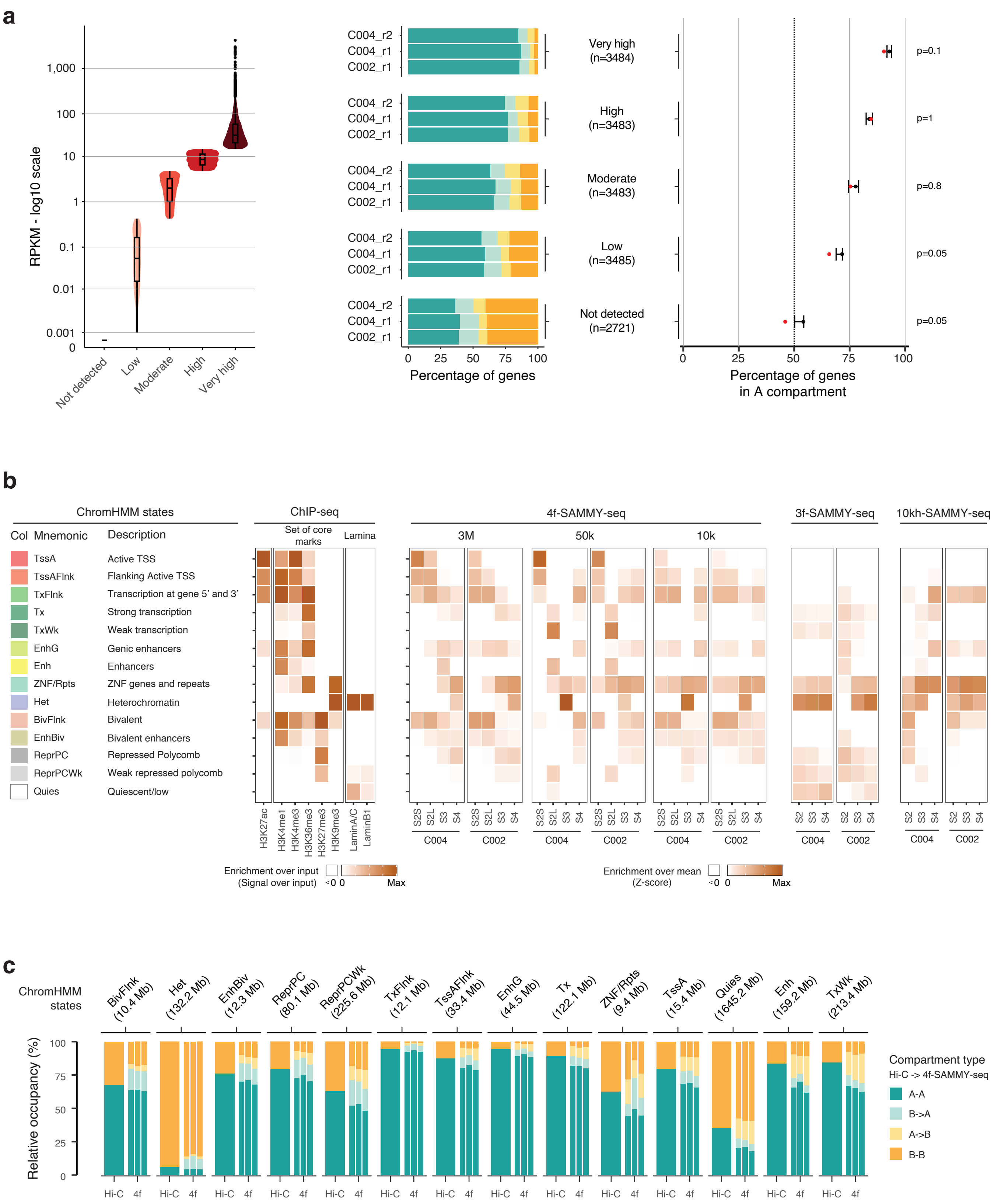


**Supplementary Figure 6 - 4f-SAMMY-seq chromatin fractions and chromatin marks association to chromatin states.**

**a)** Compartments classification comparison with gene expression status. Genes are divided by quartiles of expression level (RPKM values from Roadmap Epigenomic E055 sample) and labelled as "Low", "Moderate", "High" and "Very high" according to their expression level (violin and box plot on the left). Genes with zero RPKM (no reads as measured by RNA-seq) are assigned to the "Not detected" group. The stacked barplot shows for each 4f-SAMMY-seq replicate the relative distribution of genes across concordant and discordant compartments classifications with respect to Hi-C compartments (50kb size bins - genes assigned to bins based on the TSS position). The chromatin compartment classification is reported using the same colouring and naming convention adopted in Figure 2b. In the dot and whiskers plot on the right, we show the percentage of genes mapped in A compartment by Hi-C (red dot) and by each 4f-SAMMY-seq replicate (whiskers for maximum and minimum range, dot for the median replicate). The

significance of differences was tested with a one-sided t-test.

**b)** Epigenetic marks enrichment signatures associated to chromHMM chromatin states. We called chromatin states on a compendium of proprietary and public ChIP-seq datasets and assigned labels to maximize comparability to the chromatin states annotations adopted by the Roadmap Epigenomic consortium. Starting from the left: the colour code, the name and the description of the chromatin states, the heatmaps representing the enrichment over the input for H3K27ac, H3K4me1, H3K4me3, H3K36me3, H3K27me3 and H3K9me3, lamin A/C and B1, the heatmaps representing the enrichment over the mean (z-score) of individual SAMMY-seq fractions from protocol versions “4f” (3M, 50k and 10k), “3f” and “10kh” with two representative replicates for each of them. The histone marks originally used in the chromHMM definition of states are grouped in the "core marks" quadrant in the figure.

**c)** The stacked barplots show the percentage of regions associated with concordant ("A-A" or "B-B") and discordant ("A->B" or "B->A") compartment classification for each chromatin state (labels on the upper margin indicating chromatin states and their total size). The chromatin compartments classification is reported using the same colouring convention adopted in Figure 2b. The barplot for 4f-SAMMY-seq compartments distribution is divided in three parts to report the specific result for the three replicates (C002_r1, C004_r1 and C004_r2, respectively). The chromatin states are ordered from left to right based on the average difference between Hi-C and SAMMY-seq based compartments, as determined in Figure 3b.

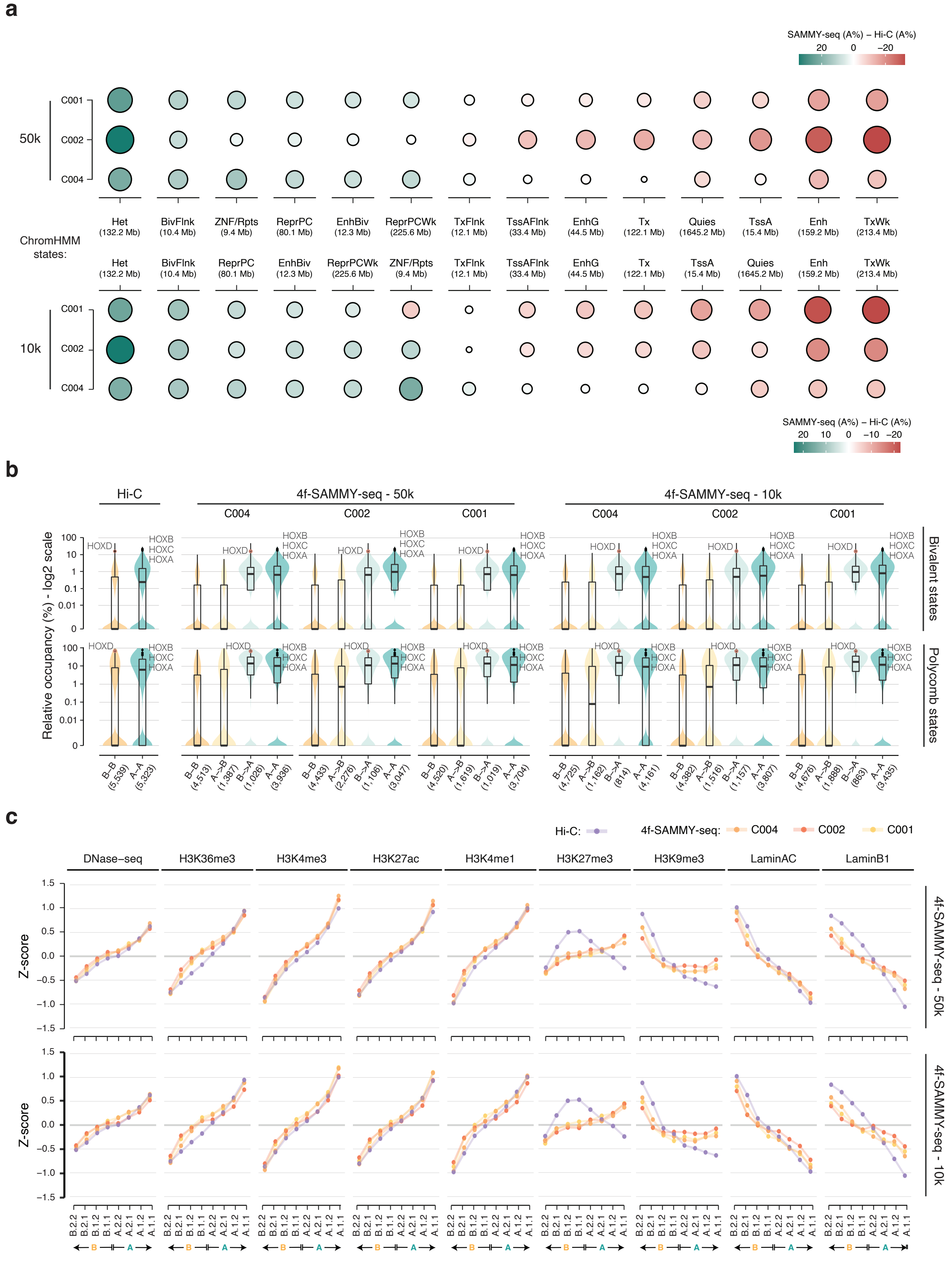


**Supplementary Figure 7 – Compartments and sub-compartments analysis on low input 4f-SAMMY-seq.**
**a)** Relative occupancy of 4f-SAMMY-seq (performed on either 50k or 10k starting cells, as indicated on the left side labels) vs Hi-C based compartments computed as the difference in "A" compartment percentage. For each chromatin state (columns, labels and total size for each state in the central row) positive values (green gradient) indicate a higher percentage of "A" compartment in 4f-SAMMY-seq based classification, whereas negative values (red gradient) indicate a higher percentage of "A" compartment based on Hi-C classification (*i.e.* higher "B" percentage based on

4f-SAMMY-seq). The size of each dot is proportional to the absolute value in the percentage difference. Chromatin states are ordered from left to right based on the percentage difference between “A” compartment in 4f-SAMMY-seq replicates (average between the three replicates, shown on the rows) and Hi-C, in a decreasing order.

**b)** Violin and box plots showing for the Hi-C dataset and for individual 4f-SAMMY-seq replicates done on either 50k or 10k cells (labels on the upper margin) the distribution of Polycomb regulated chromHMM chromatin states. The upper violin plot is for bivalent Polycomb states (EnhBiv or BivFlnk) the bottom one for monovalent repressive Polycomb chromatin states (ReprPC or ReprPCWk). Each set of violin plots shows the relative occupancy (percentage, y-axis values in $log_2$ scale) over 250kb genomic bins covering the entire genome and grouped by compartments classification ("A-A", "B-B", "A->B" or "B->A" defined as in Figure 2b) based on individual Hi-C and 4f-SAMMY-seq samples. The number of genomic bins in each group is indicated at the bottom in parentheses (x-axis). In the overlaying boxplots, the horizontal lines mark the median, the boxes margins mark the interquartile range (IQR), and whiskers extend up to 1.5 times the IQR. Specific data points associated with HOX gene clusters (black and red dots) are marked to show their positioning across groups and their associated high relative occupancy in Polycomb repressive and bivalent states.

**c)** Genome-wide mean reads enrichment in human fibroblasts (centred and scaled by chromosome, see Methods) in the eight sub-compartments classification defined by CALDER. Reads distribution profile for DNase-seq or ChIP-seq IP over INPUT enrichments are shown for sub-compartments obtained using Hi-C (purple points and lines) and 4f-SAMMY-seq data obtained from 50k and 10k starting cells (red, orange and yellow points and lines for the three replicates). Sub-compartments are sorted from the most compacted (left, B.2.2) to the most accessible one (right, A.1.1).

| | Hi-C | Low-C | 3f-SAMMY-seq | 4f-SAMMY-seq |
|---|---|---|---|---|
| Chemical modification of chromatin | YES | YES | NO | NO |
| Minimum number of starting cells | 2M | 1k | 10k | 10k |
| Chromatin compartments | YES | YES | NO | YES |
| Compartments (resolution) | 1Mb-100kb(*) | 50kb | -- | 250kb |
| Sub-compartments (resolution) | 100kb(**) | N/A | -- | 50kb |
| Raw sequencing reads or read pairs used for to call compartments (***) | 29M-3.58B | 91-517M | N/A | 102-266M |
| Time to perform protocol (****) | 3 / 4 days | 2 days | 3 hours | 3 hours |

**Supplementary Table 2: Comparison between Hi-C, 3f and 4f SAMMY-seq.** The table highlights the main characteristics and differences between SAMMY-seq and Hi-C assays. For Hi-C, the original publications about dilution Hi-C and *in situ* Hi-C protocol versions were considered (1,2): the range of values reported for number of reads and compartments resolution is referring to those in the original articles. We also report the Low-C protocol, that was designed to work on a small number of cells (3). For SAMMY protocols we compare the original 3f-SAMMY-seq (4) with the novel 4f-SAMMY-seq presented in this article. The number of sequencing reads required to call compartments are reported considering raw read counts. The reported compartments or sub-compartments resolutions are the actual analyses resolutions reported in the original cited articles and they were obtained with the number of cells and sequencing reads also reported in the table. Abbreviations used in the table: B for billion, M for million, k for thousand.

(*) Note that compartments were not defined in the original *in situ* Hi-C protocol publication (2) as it focused on sub-compartments analysis. As such, here we are reporting the sub-compartments resolution also in the compartments row for that article.
(**) Note that sub-compartments were not defined in the first Hi-C protocol (1), thus the reported value for sub-compartments is referring to the *in situ* Hi-C protocol publication (2).
(***) Note that in SAMMY-seq we used single-end reads, whereas Hi-C requires paired-end reads sequencing. Therefore, for Hi-C and Low-C experiments the actual number of sequenced reads would be twice the number reported in the table.
(****) Time to perform protocol includes library preparation in Hi-C and Low-C. In SAMMY-based protocols an additional 1.5 day is required to extract DNA and prepare libraries.

## SUPPLEMENTARY REFERENCES


1. Lieberman-Aiden, E., van Berkum, N.L., Williams, L., Imakaev, M., Ragoczy, T., Telling, A., Amit, I., Lajoie, B.R., Sabo, P.J., Dorschner, M.O. *et al.* (2009) Comprehensive mapping of long-range interactions reveals folding principles of the human genome. *Science*, **326**, 289-293.
2. Rao, S.S., Huntley, M.H., Durand, N.C., Stamenova, E.K., Bochkov, I.D., Robinson, J.T., Sanborn, A.L., Machol, I., Omer, A.D., Lander, E.S. *et al.* (2014) A 3D map of the human genome at kilobase resolution reveals principles of chromatin looping. *Cell*, **159**, 1665-1680.
3. Diaz, N., Kruse, K., Erdmann, T., Staiger, A.M., Ott, G., Lenz, G. and Vaquerizas, J.M. (2018) Chromatin conformation analysis of primary patient tissue using a low input Hi-C method. *Nat Commun*, **9**, 4938.
4. Sebestyen, E., Marullo, F., Lucini, F., Petrini, C., Bianchi, A., Valsoni, S., Olivieri, I., Antonelli, L., Gregoretti, F., Oliva, G. *et al.* (2020) SAMMY-seq reveals early alteration of heterochromatin and deregulation of bivalent genes in Hutchinson-Gilford Progeria Syndrome. *Nat Commun*, **11**, 6274.
